# Laser methane detector-based quantification of methane emissions from indoor-fed Fogera dairy cows

**DOI:** 10.5713/ab.20.0739

**Published:** 2021-01-01

**Authors:** Nobuyuki Kobayashi, Fujiang Hou, Atsushi Tsunekawa, Tianhai Yan, Firew Tegegne, Asaminew Tassew, Yeshambel Mekuriaw, Shigdaf Mekuriaw, Beyadglign Hunegnaw, Wondimeneh Mekonnen, Toshiyoshi Ichinohe

**Affiliations:** 1 Arid Land Research Center, Tottori University, Tottori 680-0001, Japan; 2 State Key Laboratory of Grassland Agro-ecosystems, Key Laboratory of Grassland Livestock Industry Innovation, Ministry of Agriculture, College of Pastoral Agriculture Science and Technology, Lanzhou University, Lanzhou, Gansu 730000, China; 3 Agri-Food and Biosciences Institute, Hillsborough, County Down BT26 6DR, UK; 4 Bahir Dar University, Bahir Dar 6000, Ethiopia; 5 College of Agriculture and Environmental Sciences, Bahir Dar University, Bahir Dar 6000, Ethiopia; 6 The United Graduate School of Agricultural Sciences, Tottori University, Tottori 680-8550, Japan; 7 Andassa Livestock Research Center, Amhara Region Agricultural Research Institute, Bahir Dar 6000, Ethiopia; 8 Faculty of Life and Environmental Science, Shimane University, Matsue 690-8504, Japan

**Keywords:** Indoor Feeding, Ethiopian Dryland, Fogera Dairy Cow, Laser Methane Detector, Methane Emissions, Napier Grass

## Abstract

**Objective:**

Portable laser methane detectors (LMDs) may be an economical means of estimating CH_4_ emissions from ruminants. We validated an LMD-based approach and then used that approach to evaluate CH_4_ emissions from indigenous dairy cows in a dryland area of Ethiopia.

**Methods:**

First, we validated our LMD-based approach in Simmental crossbred beef cattle (n = 2) housed in respiration chambers and fed either a high- or low-concentrate diet. From the results of the validation, we constructed an estimation equation to determine CH_4_ emissions from LMD CH_4_ concentrations. Next, we used our validated LMD approach to examine CH_4_ emissions in Fogera dairy cows grazed for 8 h/d (GG, n = 4), fed indoors on natural-grassland hay (CG1, n = 4), or fed indoors on Napier-grass (*Pennisetum purpureum*) hay (CG2, n = 4). All the cows were supplemented with concentrate feed.

**Results:**

The exhaled CH_4_ concentrations measured by LMD were linearly correlated with the CH_4_ emissions determined by infrared-absorption-based gas analyzer (*r*^2^ = 0.55). The estimation equation used to determine CH_4_ emissions (*y*, mg/min) from LMD CH_4_ concentrations (*x*, ppm m) was *y* = 0.4259*x*+38.61. Daily CH_4_ emissions of Fogera cows estimated by using the equation did not differ among the three groups; however, a numerically greater milk yield was obtained from the CG2 cows than from the GG cows, suggesting that Napier-grass hay might be better than natural-grassland hay for indoor feeding. The CG1 cows had higher CH_4_ emissions per feed intake than the other groups, without significant increases in milk yield and body-weight gain, suggesting that natural-grassland hay cannot be recommended for indoor-fed cows.

**Conclusion:**

These findings demonstrate the potential of using LMDs to valuate feeding regimens rapidly and economically for dairy cows in areas under financial constraint, while taking CH_4_ emissions into consideration.

## INTRODUCTION

According to the Food and Agriculture Organization of the United Nations, there are approximately 1.4 billion cattle worldwide [1], which together are a major source of greenhouse gas emissions. Indeed, 8% to 18% of greenhouse gas emissions (CO_2_ equivalent) due to anthropogenic activities are attributable to livestock farming [2]. In addition, more than 70% of the gastrointestinal methane (CH_4_, a major greenhouse gas) emissions in 2018 are attributed to cattle [3]. To reduce the greenhouse gas emissions associated with livestock farming, it will be important to develop methods of controlling the CH_4_ produced by fermentation processes in the gastrointestinal tracts of ruminants [4]. Such control will have the additional benefit of increasing livestock productivity through improved energy utilization.

In many developing countries, especially in dryland areas where cattle farming is often one of only a few viable livelihoods, cows are often grazed. However, overgrazing can result in serious soil erosion. Indeed, an estimated 73% of pasture and rangeland in the world’s drylands has been degraded, mostly as a result of overgrazing [3]. Ethiopia is one such country that is being impacted by serious soil erosion due to overgrazing; the rate of soil loss in Ethiopian rangelands (38.7 t/ha/yr) is more than five times higher than that in Ethiopian croplands (7.2 t/ha/yr) [5]. This greater soil loss in the rangelands is attributed to increased runoff resulting from intensive grazing and soil compaction [5]. To mitigate this soil loss, grazing is now restricted to areas that have little value for cropping, and indoor-fed animal production is being encouraged across the country.

To promote indoor feeding as an alternative to conventional grazing, accurate estimates of CH_4_ production from ruminants are necessary. For example, understanding enteric CH_4_ production by ruminants in different production systems is important for developing strategies to mitigate anthropogenic CH_4_ emissions [6]. Various methods for measuring CH_4_ have been developed. However, respiration chambers for open- or closed-circuit calorimetry, which are considered the gold standard for animal nutrition studies, are expensive to install and maintain [7]. The sulfur hexafluoride tracer gas technique is labor intensive and expensive with respect to changing the canisters worn around the animals’ backs and analyzing the collected samples [7]. A limitation of the GreenFeed system (C-Lock Inc., Rapid City, SD, USA) is that CH_4_ emissions cannot be measured unless the animals visit the monitoring station for feeding, and the frequency of visits may be affected by diet [8]. Therefore, cheaper and simpler methods of measuring CH_4_ with acceptable efficiency and precision are needed, especially in areas where it is financially challenging to obtain experimental equipment.

Portable laser methane detectors (LMDs) have been proposed as a potential economical means of estimating CH_4_ emissions without disturbing the normal activities of cattle. In this application, the device emits a laser beam that is directed at an animal’s nostril; the device then automatically measures the CH_4_ concentration (ppm m) along the length of the beam [9]. In a validation study, the CH_4_ concentrations in the exhaled air of 72 steers, as measured by LMD, were correlated with the concentrations measured by using respiration chambers (*r*^2^ = 0.39, p<0.01) [10]; in this study, the LMD values were obtained after first measuring the CH_4_ emissions in respiration chambers using the same animals. Another study reported the use of LMD on a farm to determine CH_4_ concentrations in the breath of 622 dairy cows; however, the values determined by LMD were not validated against another method [11]. Thus, further validation of the LMD approach by using values recorded simultaneously by means of an already validated method (*e.g*., respiration chambers) is needed before LMDs can be applied in feeding trials examining CH_4_ emissions.

Here, we examined the use of an LMD-based approach to estimate CH_4_ emissions through two *in-vivo* experiments for cattle. First, we validated our LMD-based approach against a respiration chamber-based approach in Simmental crossbred beef cattle (Exp 1). Then, we performed a feeding trial to examine the effects of indoor feeding on the CH_4_ emissions and lactation performance of Fogera dairy cows (Exp 2).

## MATERIALS AND METHODS

### Animal care

The cattle used in this study were treated according to the Tottori University provisions and regulations for animal experiments throughout all the experimental periods, under approval from the Committee of Animal Experiments of Tottori University (No. 20-T-17).

### Validation of CH_4_ emissions estimated by LMD against those measured by infrared-absorption-based gas analyzer in an indirect open-circuit respiration calorimeter chamber (Exp 1)

The CH_4_ emissions of cattle were estimated by both respiration chamber and LMD. Because of a lack of respiration chambers in Ethiopia (the site for Exp 2), we performed this experiment at Linze Grassland Agriculture Trial Station (39.24°N, 100.06°E), Lanzhou University, China, using two Simmental crossbred male beef cattle (not castrated; body weight [BW], 224 and 260 kg; age, 9 mo). The experimental period was 12 d (17 to 28 Sept 2019). Each animal was provided one of two diets throughout the experimental period: a high-concentrate diet (HC) comprising alfalfa hay (1.1 kg-dry matter [DM]/d), wheat straw (1.1 kg-DM/d), and commercial concentrate feed (1.5 kg-DM/d), or a low-concentrate diet (LC) comprising the same feed ingredients but at 2.5, 2.5, and 0.8 kg-DM/d, respectively (Supplementary Tables S1 and S2). Both diets were designed to provide the net energy and crude protein required for a bull to gain 1 kg BW daily on the basis of the estimation equation and tabular values of feed ingredients presented in Feeding Standard for Beef Cattle [12]. The daily DM intakes of roughage (alfalfa hay and wheat straw) and of the concentrate feed were recorded for each animal throughout the experimental period.

After 5 d in cubicle accommodation (*i.e*., on d 6 after the start of cubicle accommodation), each animal was transferred to an indirect open-circuit respiration calorimeter chamber (chamber capacity, 17.8 m^3^) for 7 d (4 d for adaptation and 3 d for measurement). The CH_4_ concentration in the exhaust air from each chamber was measured every 15 min for 48 h by using an infrared-absorption-based gas analyzer (VA-3000, Horiba Ltd., Kyoto, Japan). The air temperature and humidity in the chamber were recorded continuously and remained in the range from 12.2°C to 25.5°C and from 17.9% to 56.7%, respectively. Air influx in each chamber adjusted for the gas volume under standard conditions was recorded. On d 10, samples of the feed ingredients were collected to determine the concentrations of ash-free neutral detergent fiber (NDFom).

While the cattle were in the respiration chamber, CH_4_ concentrations were measured simultaneously by using both the gas analyzer and an LMD (SA3C32B, Tokyo Gas Engineering Co. Ltd., Tokyo, Japan) for two 12-h periods from 18:00 to 06:00. The LMD instrument uses a non-visible laser and infrared-absorption spectroscopy to measure the CH_4_ concentration (LMD-CH_4_) at 0.5-s intervals. The wavelength of the infrared ray is fixed at 1,653 nm, which corresponds to the absorption line of CH_4_.

LMD-CH_4_ was measured in the respiration chamber with the LMD held at a distance of 0.6 to 1.2 m from the animal’s nostrils. However, the frequent movement of cattle during the day made it difficult to accurately aim the LMD. A preliminary experiment prior to Exp 1 revealed that the average CH_4_ emissions (mg-CH_4_/min) of four cattle in the respiration chambers over two 23-h periods (each from 07:00 to 06:00) were highly correlated with the average values for the 12-h period from 18:00 to 06:00. The average CH_4_ emissions over 23 h (*y*, mg-CH_4_/min) were therefore estimated from those over 12 h (*x*, mg-CH_4_/min) with *y* = 1.072*x*–1.891 (*r*^2^ = 0.95). A similar correlation has been reported for eight steers in respiration chambers between 24-h and nocturnal (00:00 to 06:30) heat-production values (*r*^2^ = 0.81 to 0.90), and between 24-h heat-production and CH_4_ emissions (*r*^2^ = 0.55 to 0.66) [13]. Therefore, we assumed that the CH_4_ emissions measured at night would provide acceptable estimation of 24-h CH_4_ emissions.

LMD-CH_4_ was measured once an hour during the night for two 12-h periods (18:00 to 06:00) for each animal. Each of the LMD-CH_4_ measurement took less than 5 min, and 2 to 3 min of data per measurement were used after eliminating data not usable for analysis (*e.g*., data where the LMD was not pointing exactly at the nostril). Entry of a person into the chamber for the purpose of taking measurements was assumed to have minimal effects on the animals. Nevertheless, to reduce the effects of the person’s entry, we kept the doors of respiration chambers closed after the entry for each measurement, and standardized the length of time spent in the respiration chamber for each measurement.

In the preliminary LMD-CH_4_ datasets, two trends in the LMD-CH_4_ values were observed, one for eructation and another for respiration; this is consistent with a previous report [10]. Therefore, assuming a double normal distribution, each hourly LMD-CH_4_ dataset was split into two sub-datasets, one for eructation and one for respiration. A total of five statistical parameters were calculated for each dataset: the mean and the standard deviation for the LMD-CH_4_ values within each of the two sub-datasets and the ratio distribution for the two sub-datasets that achieved the highest likelihood. For the calculation of these five parameters, the nonlinear generalized reduced gradient solving (nonlinear GRG) method in Excel 2019 (Microsoft Corporation, Redmond, WA, USA) was used. The higher LMD measurements were assumed to represent CH_4_ emissions by eructation, whereas the lower LMD measurements were considered to represent CH_4_ emissions by respiration.

Then, the two probabilities for a single LMD-CH_4_ value, namely one in the normal distribution for respiration and the other in the normal distribution for eructation, were calculated. Each LMD-CH_4_ value was then categorized according to these probabilities into one of two sub-datasets (for eructation or for respiration) (Supplementary Figure S1). The LMD-CH_4_ datasets that could not be clearly separated into the two sub-datasets (*i.e*., dataset with a low power for the test for eructation and respiration) were excluded. Of the 42 LMD-CH_4_ datasets collected from the two cattle, 34 could be separated into two normal distributions, one each for respiration and eructation. The statistical power of the test for each of the 34 datasets ranged from 72.8% to 94.8%.

Each of the 34 datasets contained three mean values: ones for the two sub-datasets (for respiration and eructation) and the other for the combined sub-datasets (before their separation into respiration and eructation). Furthermore, three mean-value groups were obtained: the first group composed of 34 mean values for the 34 sub-datasets for respiration, the second group for the 34 sub-datasets for eructation, and the third group for the 34 datasets before separation into respiration and eructation. Each of the three mean-value groups was then regressed by using the least-squares method against the dataset obtained from the respiration chamber measurements.

During the analysis, we observed time delays for when the values obtained by the LMD were reflected in the values recorded by the gas analyzer. These delays were probably related to the distance from the respiration chamber to the gas analyzer, which was in the general control room. Therefore, we calculated correlation coefficients for each of the three mean-value groups and each of six datasets obtained with the gas analyzer at 0, 15, 30, 45, 60, and 75 min after the LMD-CH_4_ measurement. The correlation coefficients were calculated by using R statistical software (version 3.1.1, R Foundation for Statistical Computing, Vienna, Austria). By using the pair of datasets with the highest correlation coefficient, an equation to estimate daily CH_4_ emissions using the nocturnal LMD values was formulated.

### Comparison of CH_4_ emissions from grazing versus indoor-fed dairy cows (Exp 2)

A feeding trial for indigenous cows (Fogera breed) was performed for 24 d (from 21 Aug to 13 Sept 2019) at Andassa Livestock Research Center, Ethiopia (11.42 to 11.92°N, 37.07 to 37.65°E; elevation, 1,730 to 1,750 m above sea level). This center recently received 1,434 mm of annual rainfall, and the average daily temperature ranged from 8.8°C (in Jan) to 29.5°C (in Mar) (data supplied by the Andassa Research Center). Twelve multiparous (2 or 3 parity) dairy cows (mean BW, 227.4±23.1 kg) in midlactation (107±27 d in milk at the start of Exp 2) were allocated into one of three feeding groups: a grazing group (GG, n = 4; control) and two indoor-feeding groups fed with natural-grassland hay (CG1, n = 4) or with Napier-grass (*Pennisetum purpureum*) hay (CG2, n = 4).

The natural-grassland hay used as the feed for CG1 was purchased from a private dairy farm and was composed mainly of *Andropogon*, *Cynodon*, *Digitaria*, *Hyparrhenia*, and *Panicum* spp. as well as *Trifolium quartinianum*, T*rifolium polystachyum*, and *Indigofera atriceps*. In addition to these species, *Trifolium subterraneum* and *Eleusine indica* were observed on the grazing land of the research center used for GG. Napier grass was also examined because it was widely available and was assumed to be a major forage in the drylands of Ethiopia owing to its high DM yield (18 to 23 t-DM/ha/yr) [14] and high crude-protein content (15.8% DM) [15]. The Napier grass was harvested from irrigated land at the research center and air dried in the field for at least 3 d before use.

All three diets were designed to provide sufficient net energy and crude protein for a 3-kg daily milk yield by using the BW of the cows, the estimation equation presented in Nutrient Requirements of Dairy Cattle [16], and reported nutrient concentrations of the feed ingredients [17]. For the GG cows, natural-grassland hay, Napier grass, and concentrate were offered, respectively, at 0.0, 0.0, and 1.5 kg-DM/d; for the CG1 cows at 3.2, 0.0, and 1.5 kg-DM/d; and for the CG2 cows at 0.0, 3.8, and 1.5 kg-DM/d. The GG cows were expected to graze similar amounts of natural-grassland hay as the CG1 cows.

The daily feed allowance for each cow was adjusted on the basis of BW at the start of the experiment. Throughout the experimental period, the GG cows were allowed to graze daily from 8:00 to 16:00 and were accommodated indoors during rest hours; no roughage (natural-grassland hay or Napier grass) was provided when the cows were accommodated indoors. The CG1 and CG2 cows were provided with natural-grassland hay and Napier grass, respectively, twice a day (at 08:00 and 17:00). The roughage for CG1 and CG2 was chopped into 5- to 10-cm lengths for feeding. The feed for all the groups was supplemented with concentrate feed when the cows were milked twice a day at 07:00 and 16:00. The concentrate consisted (on a DM basis) of maize grain (40%), Noug seed cake (49%), wheat bran (8%), salt (1%), and ruminant premix (2%; Intraco Ltd., Antwerp, Belgium). All the cows were offered water twice a day during the daytime.

As described for Exp 1, LMD-CH_4_ values were recorded for each cow each hour for 2 nights (*i.e*., two periods of 18:00 to 06:00) after the adaptation period had passed (from d 6). Of the 286 datasets of hourly LMD-CH_4_ measurements from the 12 cattle, 263 could be separated into two normal distributions for respiration and eructation. The statistical power of the test for eructation and respiration in each of the 263 datasets ranged from 75.3% to 98.1%. By using the regression equation obtained in Exp 1, the mean value of each of the three mean-value groups—for eructation, respiration, or both—was converted into a daily CH_4_ emission for each cow.

The weight of feed offered and refusals were recorded daily throughout the experimental period to calculate daily feed intake. Samples of the feed ingredients (grazing herbage, natural-grassland hay, Napier grass, and concentrate) were collected for chemical analysis on d 17. The BW of each cow was recorded at the start and end of the experiment, and on the days of LMD measurement. Daily milk yields (summation of both the morning milking and afternoon milking) were measured throughout the experimental period.

To examine the fecal excretions and determine digestive coefficients for all of the cows, spot fecal samples (about 500 g/sample) were collected three times a day from d 17 to 21 and stored at −15°C until analysis. In addition, to estimate the DM intake for the four GG cows, 2.5 g of ground chromium oxide (Cr_2_O_3_) was mixed with the concentrate feed provided twice a day, from d 12 to 18, and again spot samples fecal were collected.

The feed and fecal samples were dried at 105°C in a forced-air oven for more than 6 hours to constant weight and ground to pass through a 1-mm screen. Then, by using the standard methods of the Association of Official Analytical Chemists [18], the concentrations of crude protein (method no. 984.13), ether-extracted fat (crude fat; 920.39), ash-free acid detergent fiber and acid detergent lignin (973.18), and crude ash (942.05) in the dried feed and fecal samples were determined. The concentration of NDFom was determined as reported elsewhere [19]. Fecal Cr_2_O_3_ concentrations were also determined as reported elsewhere [20], and the weight of fecal excretions of the GG cows were estimated. The DM digestive coefficients of all the cows were then calculated by using the acid detergent lignin concentrations in the feed and fecal samples as internal markers. We used the DM digestive coefficients and the weight of fecal excretions to calculate the DM intake of GG cows.

Two estimates of CH_4_ emissions were calculated by using the following equations reported by Niu et al [21] and Hristov et al [22], respectively:

$${\text{CH}}_{4}\;\text{emissions }(\text{g}/\text{d})=13.3\times \text{DM intake }(\text{kg}/\text{d})+124$$

$$\begin{array}{l} {\text{CH}}_{4}\;\text{emissions }(\text{Mcal}/\text{d})\\ =0.0392\times \text{gross-energy intake }(\text{GEI},\text{Mcal}/\text{d})\\ +0.0189\times \text{NDFom concentration }(\%)\\ -0.156\times \text{ether-extracted fat concentration }(\%)\\ +0.0014\times \text{BW }(\text{kg})+0.37 \end{array}$$

The GEI value used in the equation of Hristov et al [22] was calculated by using an equation reported elsewhere [23]. These two estimates were compared with the CH_4_ emissions recorded by the gas analyzer in Exp 1 and with those estimated by using the LMD in Exp 2.

Each of the datasets obtained in Exp 2 was analyzed by using the model *y_ij_* = *μ*+*α_i_*+*ɛ_ij_*, where *y_ij_* is the dependent variable, *μ* is the overall mean value for each dataset, *α_i_* is the fixed effect of treatments (feeding style and ingredients), and *ɛ_ij_* is the random residual error of the *j*th cow with the *i*th treatment. Differences in means among the three groups were tested by using one-way analysis of variance. When the treatment effect was significant (p<0.05), multiple comparisons were tested using Tukey’s method. These statistical analyses were performed with R statistical software (version 3.1.1, R Foundation for Statistical Computing, Vienna, Austria).

## RESULTS

### Experiment 1

**Figure 1:**
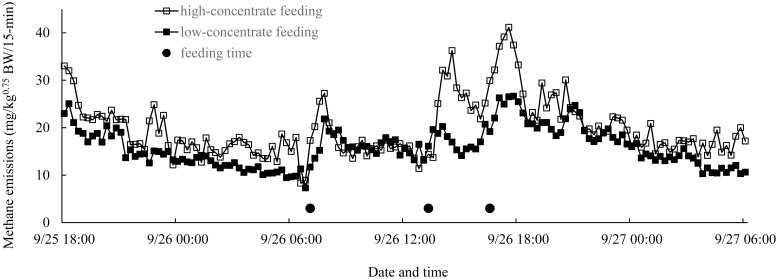
Methane emissions of Simmental crossbred beef cattle in respiration chambers, as determined by infrared-absorption-based gas analyzer (Exp 1). □, cow (body weight, 224 kg) fed high-concentrate diet; ■, cow (body weight, 260 kg) fed low-concentrate diet; ●, feeding time. Methane emissions increased immediately after feeding.

For both cattle, the diets were almost all consumed shortly after the start of feeding. The daily intake of feed and nutrients, and the ratio of concentrate-feed intake to total DM intake are shown in Table 1. The amounts of NDFom in the alfalfa hay, wheat straw, and concentrate feed were 52.7%, 77.2%, and 22.8%-DM, respectively. Gas analysis revealed that CH_4_ emissions increased immediately after feeding (Figure 1). The CH_4_ emissions (mg/kg^0.75^ BW/15-min) at the three feeding times between the two nocturnal LMD-measuring periods were 17.3, 14.4, and 29.9 for the cattle fed HC, and 11.7, 16.1, and 19.2 for the cattle fed LC. In addition, the peak CH_4_ emissions after each of the three feedings were 27.2, 36.2, and 41.1 for the cattle fed HC, and 21.8, 20.2, and 26.3 for the cattle fed LC. The average daily CH_4_ emission was 1.91 and 1.53 g/kg BW^0.75^ for the cattle fed the HC and LC diets, respectively (Table 1). The CH_4_ emissions (mg/kg^0.75^ BW/15-min) of the cattle fed the HC diet were higher than those of the cattle fed the LC diet at 123 of the total of 145 datapoints (Figure 1).

**Figure 2:**
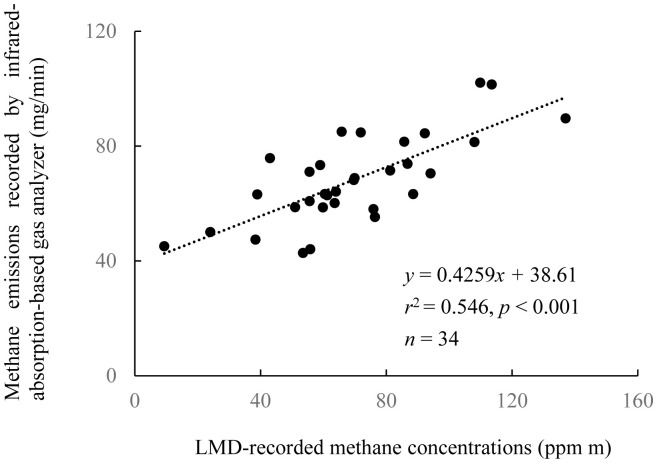
Linear regression of methane concentrations recorded by laser methane detector (LMD, *x*) versus average methane emissions recorded by infrared-absorption-based gas analyzer for 60 min after LMD measurement (*y*) in Exp 1. For both measurements, the cattle (Simmental beef cattle) were held in respiration chambers. LMD-recorded methane concentration (*x*) of each datapoint was the mean value of methane concentrations, which were considered to represent methane emissions by respiration in each hourly measurement.

The mean-value group comprising the respiration sub-datasets (*x*, ppm m) was most significantly correlated with the gas analyzer dataset up to 60 min after the LMD-CH_4_ measurement (*y*, mg-CH_4_/min; Table 2). Using these two datasets, the regression equation was *y* = 0.4259*x*+38.61 (*r*^2^ = 0.55, p<0.001, Figure 2).

### Experiment 2

The NDFom concentration in the Napier grass used in the CG2 diet was lower than that in the natural-grassland hay used in the CG1 diet and that of the grazing grasses (Table 3); therefore, the NDFom concentration of ingested feed was lower in CG2 than in CG1 and GG (Table 4). The crude-protein concentration in the Napier grass was higher than that in the natural-grassland hay and the grazing grasses (Table 3), and the crude-protein intake in CG2 was the highest among the three groups (Table 4). The calculated GEI was 21.1, 18.5, and 22.0 Mcal/d for GG, CG1, and CG2, respectively. DM intake was comparable between GG and CG2 (p = 0.13), and GG and CG1 (p = 0.09), but it was significantly lower in CG1 than in CG2 (p<0.01, Table 4). NDFom intake was lower in CG1 than in GG (p<0.01), whereas the ratio of concentrate intake to total DM intake was higher in CG1 (p = 0.01) and lower in CG2 (p = 0.05) than in GG. DM digestibility was lower in CG1 than in GG (p<0.01), whereas NDFom digestibility was lower in CG1 and CG2 than in GG (both p<0.01). Body-weight gain was higher in CG2 than in CG1 (p = 0.03) and was negative in CG1. The estimated CH_4_ emissions (g/kg^0.75^ BW/d) did not differ among the three groups (Table 4). CH_4_ emissions per milk yield did not differ among the groups (p = 0.95). However, CH_4_ emissions per DM intake and the ratio of CH_4_ emissions to estimated GEI were significantly higher in CG1 than in the other groups (both p<0.05).

## DISCUSSION

### Correlation of CH_4_ emissions estimated by LMD with those measured by gas analyzer in a respiration chamber

In Exp 1, prompt increases in CH_4_ emissions after feeding were detected by the gas analyzer (HC 17.3–29.9 to 27.2–41.2, LC 11.7–19.2 to 20.2–26.3 mg/kg BW^0.75^/15-min; Figure 1) and were consistent with previously reported values for CH_4_ emissions during feeding (15 to 50 mg/kg^0.75^ BW/15-min) [24].

More than 80% of the hourly measurement datasets could be used to produce the two normal distributions, indicating that the measurement duration (<5 min) used here, which was similar to that used in previous studies (4 min [10], <5 min [25]), was appropriate. A previous study reported that the total time spent in eructation as a percentage of the total LMD-measurement time ranged from 28.7% to 49.4% [10]. In our study, the percentage of LMD-CH_4_ values categorized into eructation in each of the LMD-CH_4_ datasets—which could be regarded as the percentage of total time spent in eructation—ranged from 11.7% to 48.3% (Exp 1). Our results thus appeared to be consistent with those of the previous study [10]. Each LMD-CH_4_ value in the LMD-CH_4_ dataset could be properly categorized into one of the two sub-datasets for eructation and respiration.

For all the periods (after LMD measurement) used for the regression, the respiration sub-datasets were correlated more significantly than the eructation datasets with the CH_4_ emissions dataset determined with the gas analyzer (Table 2). Although more than 80% of the CH_4_ exhaled by cattle is associated with eructation [10], the higher correlation coefficients obtained here by using the respiration sub-datasets indicated that respiration was more useful than eructation for quantifying the CH_4_ emissions of individual cattle. Moreover, the respiration sub-datasets were well correlated with the gas-analyzer dataset for 0 to 60 min after LMD-CH_4_ measurement (Table 2, Figure 2). The time delay until when the values obtained by the LMD were reflected in the values recorded by the gas analyzer was thus estimated as 60 min. The correlation coefficient (up to 60 min, *r*^2^ = 0.55) was higher with this dataset than it was with the datasets obtained for the final 45 min (15 to 60 min, *r*^2^ = 0.46), 30 min (30 to 60 min, *r*^2^ = 0.46), and 15 min (45 to 60 min, *r*^2^ = 0.42) periods after LMD measurement. The decrease in *r*^2^ value with increasing time after LMD measurement suggested that diffusion of CH_4_ exhaled by the animal started affecting the values recorded by the gas analyzer immediately after the LMD-CH_4_ measurement.

A previous study [10] reported three equations with high goodness of fit for the estimation of CH_4_ emissions, using three statistical parameters obtained from each LMD dataset: the ratio of total time spent in eructation to total measurement time, the maximum CH_4_ concentration (ppm m) during the total time spent in respiration, or a combination of both parameters. We calculated correlation coefficients for each of these three parameters for the CH_4_ emissions dataset obtained for 60 min (0 to 60 min) after LMD-CH_4_ measurement. However, all three correlation coefficients calculated (*r*^2^<0.01, = 0.07, and = 0.44, respectively) were lower than the coefficients demonstrated in our study (*r*^2^ = 0.55).

Ultimately, we constructed an equation to estimate the CH_4_ emissions in Exp 2 on the basis of the CH_4_ emissions for 0 to 60 min after LMD-CH_4_ measurement and the LMD-CH_4_ respiration dataset. The resulting estimation equation was *y* = 0.4259*x*+38.61 (*y*, CH_4_ concentration [mg/min]; *x*, mean of respiration sub-datasets recorded by LMD [ppm m]).

### Effects of grazing versus indoor feeding on productivity and CH_4_ emissions of dairy cows

Previously, at the site of Exp 2, the crude-protein concentration (on a DM basis) in Napier grass was reported as 9.3% [17]. In our study, the crude-protein concentration in the Napier grass was 8.2% (Table 3). In these two studies, the agronomic practices for Napier grass production followed those previously recommended for this grass (International Livestock Research Institute accession number 15743) [26]. A crude-protein concentration of at least 8% (on a DM basis) is needed if forage is given as a sole diet to ruminants [27]. The higher Napier grass crude-protein concentrations in both our study and the former study (9.3% [17]) than in the latter study (8% [27]) indicated that this grass could be used as a basal diet for Fogera dairy cows. By contrast, the crude-protein concentration in natural-grassland hay in our study (4.5%; Table 3) was lower than that in the Napier grass (8.2%). The concentrations of the crude protein and NDFom (72.1%) in the natural-grassland hay were consistent with those in the previous study (crude protein, 4.2%; NDFom, 74.2% [17]). These findings appeared to suggest that natural-grassland hay could not be used as a basal diet for the Fogera dairy cows, and that a concentrate supplement would be needed if natural-grassland hay were fed as the basal forage.

The similarity in the CH_4_ emissions per metabolic body size and in the ratios of emitted-CH_4_ energy to GEI observed between the cows in GG and CG2, and the numerically (albeit not significantly) higher milk yield and BW gain in CG2 than in GG (Table 4) suggested that Napier grass was a suitable forage for indoor feeding. The CH_4_ emissions per milk yield in CG2 and GG were also comparable (46.8 g/L-milk vs 49.3 g/L-milk; p = 0.97).

The concentration of dietary fiber (*i.e*., NDFom) affects voluntary feed intake through physical regulation via rumen fill [28]. Less fibrous diets with low NDFom concentrations promote dietary passage through the rumen and increase DM intake and decrease digestibility [28]. This may explain the slight increase in DM intake but decrease in DM digestibility in CG2 compared with in GG (Table 4). Likewise, the significantly lower DM digestibility in CG1 than in GG (p<0.01) was accompanied by a significantly lower dietary NDFom concentration and a significantly higher ratio of concentrate intake to total DM intake (both p<0.05). The lack of a significant difference in DM intake between CG1 and GG (p = 0.09) and the slightly higher CH_4_ emissions in CG1 than in GG (p = 0.20) resulted in significantly higher CH_4_ emissions per DM intake in CG1 (p<0.01). Nevertheless, milk yield (L/d) did not differ between GG and CG1 despite the lower DM digestibility in CG1 than in GG.

Daily BW gain was insignificant in CG1 but positive in the other groups. The metabolizable energy required for maintenance of indoor-fed cows of 600-kg BW is 7% less than that of cows grazing for 2 h/d [16]. The GEI decrease in CG1 compared with GG was 12.5%, although the DM intake did not significantly differ between CG1 and GG (p = 0.09). The GEI decrease in CG1 was more than the difference between the energy intakes required for grazing and for indoor-feeding. By contrast, the milk yield was comparable between CG1 and GG (p = 0.81). Milk yields in all three groups were less than 3 L/d (the milk-yield target). The GEI decrease in CG1 compared with GG—which was more than the decrease acceptable for BW gain—and the comparable milk yield (albeit both less than the target yield) between CG1 and GG might have led to the lack of BW gain in CG1. Together with the significantly lower DM digestibility in CG1 than in GG, these findings suggest that natural-grassland hay is not a suitable feed for indoor feeding. The lower CH_4_ emissions per DM intake in CG2 than in CG1 were consistent with the findings of a previous report [17], and with those of another report that demonstrated that a less fibrous diet with a low NDFom concentration (*i.e*., CG2) decreases CH_4_ emissions [29].

### Assessment of the equation obtained by using CH_4_ concentrations recorded by LMD to estimate CH_4_ emissions

The high statistical powers of the test used to separate the LMD-CH_4_ dataset into eructation and respiration sub-datasets in both experiments, and the fact that the respiration sub-datasets were moderately well correlated with the CH_4_ emissions dataset collected by the gas analyzer in Exp 1 (Figure 2), indicated that the maximum likelihood estimation was appropriate for obtaining a group of datasets that could be used to provide an equation to estimate CH_4_ emissions.

The CH_4_ emissions per metabolic body size estimated in Exp 1 (Table 1) and Exp 2 (Table 4) were lower than those calculated by using the equations of Niu et al [21] (HC [Exp 1], 2.64; LC [Exp 1], 2.98; GG [Exp 2], 3.15; CG1 [Exp 2], 3.10; CG2 [Exp 2], 3.22 g/kg^0.75^ BW/d) and Hristov et al [22] (HC [Exp 1], 2.33; LC [Exp 1], 3.37; GG [Exp 2], 2.93; CG1 [Exp 2], 2.70; CG2 [Exp 2], 2.82 g/kg^0.75^ BW/d). These gaps could not be eliminated by recalculation of the CH_4_ emissions by using the correlation between those for 23 h (*y*) and for nocturnal 12 h (*x*) as revealed in our preliminary experiment (*y* = 1.072*x*–1.891). Nevertheless, the ratios of CH_4_ emissions to estimated GEI in Exp 2 (4.1% to 5.0%) were consistent with previously reported ratios (2% to 15% [30]). The NDFom concentrations of the ingested diets in Exp 1 (LC, 59%; HC, 48%) and Exp 2 (GG, 65.5%; CG1, 60.4%; CG2, 59.3%) were higher than those used by Niu et al [21] and Hristov et al [22] for constructing their equations (35.4%±7.66% and 34.3%± 7.47%, respectively). In contrast, DM intake in both of our experiments (Exp 1, 3.7 to 5.6 kg-DM/d; Exp 2, 4.20 to 4.93 kg-DM/d) was lower than those used by Niu et al [21] and Hristov et al [22] for constructing their equations (18.5±4.60 kg-DM/d and 16.5±4.30 kg-DM/d, respectively). The lower CH_4_ emissions that we obtained, despite the use of high fiber diets in all of the groups, might have been due to the inappropriate extrapolation of values by the previously reported equations, which were constructed by using datasets of cattle breeds different from those used here (Holstein, Ayrshire, Jersey, Brown Swiss [21]; Holstein, Jersey, Angus, Hereford [22]).

Here, we report several findings. First, the equation that we obtained to estimate CH_4_ emissions (y, mg/min) from LMD CH_4_ concentrations (*x*, ppm m) was *y* = 0.4259*x*+38.61 (*r*^2^ = 0.55). We also observed no difference in the CH_4_ emissions in CG2 compared with in GG, suggesting that Napier grass is a suitable feed for indoor feeding, and this finding was supported by the preferable milk yield in CG2. Moreover, we demonstrated that LMDs can be used to test feeding regimens with consideration of the milk productivity and CH_4_ emissions of dairy cows. Feeding regimens to both increase productivity and reduce greenhouse-gas emissions (*i.e*., to improve energy utilization) are important, especially in areas under financial constraints to feed cows with commercial concentrate feeds. The use of LMDs will make conducting feeding trials cheaper and simpler than using the other methods currently available for determining CH_4_ concentrations. This will be useful for studies conducted in such financially challenged areas, particularly in developing countries.

## Supplementary Information



## Figures and Tables

**Table 1:** Feed and nutrient intake, methane emissions, and daily body-weight gain of two Simmental crossbred beef cattle fed a high- or low-concentrate diet (Exp 1)

Items	HC	LC
Feed and nutrient intake		
Roughage (kg-DM/d)	2.2	4.8
Concentrate (kg-DM/d)	1.5	0.8
Ash-free neutral detergent fiber (kg/d)	1.8	3.3
Ratio of concentrate intake to total DM intake (%)	40.0	13.9
Methane emissions		
g/kg^0.75^ BW/d	1.91	1.53
g/kg-DM intake/d	30.2	17.1
Daily BW gain (kg/d)	0.43	1.21

Alfalfa hay and wheat straw mixed 50:50 on a DM basis for roughage.

HC, high-concentrate diet; LC, low-concentrate diet; DM, dry matter; BW, body weight.

**Table 2:** Correlation coefficients between the infrared-absorption-based gas analyzer and laser methane detector datasets in Exp 1

Items	Respiration	Eructation	Overall
Period used for regression after the LMD measurement, min			
0	0.1957^**^	0.1158	0.1213^*^
0 to 15	0.2250^**^	0.1767^*^	0.1759^*^
0 to 30	0.3401^***^	0.1225^*^	0.1709^*^
0 to 45	0.4817^***^	0.2254^**^	0.2999^**^
0 to 60	0.5463^***^	0.2224^**^	0.3038^**^
0 to 75	0.4532^***^	0.1663^*^	0.2216^**^

LMD, laser methane detector.

The period used for regression after the laser methane detector measurement is the gas analyzer measurement period: for example, “0 to 15” means that the gas analyzer data for the 15-min period after LMD measurement were used to calculate correlation coefficients.

Significance of correlation coefficient;

* 0.01≤p<0.05,

** 0.001≤p<0.01,

*** p<0.001.

**Table 3:** Chemical compositions of the feed ingredients used in Exp 2

Feed	Chemical composition (% DM)					

	CP	EE	NDFom	ADFom	ADL	CA
Natural-grassland hay	4.5	1.5	72.1	48.2	8.3	11.1
Napier grass	8.2	1.8	68.3	42.9	6.7	11.9
Concentrate	19.4	5.9	34.1	18.6	3.5	10.3
Grazed grass	2.5	1.7	77.8	46.5	5.9	7.0

DM, dry matter; CP, crude protein; EE, ether-extracted fat; NDFom, ash-free neutral detergent fiber; ADFom, ash-free acid detergent fiber; ADL, acid detergent lignin; CA, crude ash.

**Table 4:** Feed and nutrient intake, digestibility, milk yield, methane emissions, and body-weight gain in Fogera dairy cows (Exp 2)

Items	GG^1)^	CG1^1)^	CG2^1)^	SEM	p-value
Feed and nutrient intake					
DM (kg/d)	4.59^ab^	4.20^b^	4.93^a^	0.226	0.004
Crude protein (kg/d)	0.33^c^	0.38^b^	0.55^a^	0.024	<0.0005
Crude-protein concentration (%)	7.21^c^	9.04^b^	11.1^a^	0.148	<0.0005
NDFom (kg/d)	3.00^a^	2.54^b^	2.93^a^	0.137	0.002
NDFom concentration (%)	65.5^a^	60.4^b^	59.3^c^	0.409	<0.0005
Ratio of concentrate intake to total DM intake (%)	28.1^b^	30.7^a^	26.2^c^	0.992	<0.0005
Digestibility					
DM (%)	58.6^a^	46.4^b^	50.8^ab^	3.990	0.006
NDFom (%)	72.8^a^	54.8^b^	61.3^b^	4.292	0.0007
Milk yield (L/d)	1.33	1.56	1.64	0.478	0.70
Milk yield (L/kg-DM intake/d)	0.29	0.37	0.33	0.103	0.61
Methane emissions					
g/d	65.9	69.5	66.1	2.927	0.17
g/kg^0.75^ BW/d	1.12	1.20	1.12	0.092	0.44
Methane-energy/GEI (%)	4.14^b^	5.00^a^	3.99^b^	0.205	<0.0005
Body-weight gain (kg/d)	0.25^ab^	−0.07^b^	0.55^a^	0.273	0.03

Data for milk yield of one cow in GG could not be collected; therefore, the available data were used (n = 3).

SEM, standard error of means; DM, dry matter; NDFom, ash-free neutral detergent fiber; BW, body weight; GEI, gross-energy intake.

1) Experimental diets: GG, grazing group (control); CG1, indoor natural-grassland-hay feeding; CG2, indoor Napier-grass feeding.

a,b,c Means in the same row with different superscripts differ significantly (p≤0.05).
