# Supplementary material for: Laser methane detector-based quantification of methane emissions from indoor-fed Fogera dairy cows
Source: Anim Biosci. 2021 Jan 1;34(8):1415–24. doi: 10.5713/ab.20.0739 (PMC8255885; doi:10.5713/ab.20.0739)
Supplement: Supplementary file 1 [file ab-20-0739-suppl.pdf]

Supplementary Table S1. Energy and crude-protein concentration of feed ingredients in the diets for the cattle used in Exp 1

Item	Net-energy concentration (MJ/kg-DM)		Crude protein (% DM)
	Maintenance (NEm)	Gain (NEg)	
Alfalfa hay	4.60	1.09	13.0
Wheat straw	3.97	0.00	3.0
Concentrate[1]	8.08	6.15	20.7
Corn grain	9.39	7.21	8.5
Wheat bran	6.95	4.50	14.3
Cottonseed meal	7.35	6.49	43.5
Soybean meal	8.71	6.20	44.2
Stone powder	-	-	0.00
Calcium hydrogen phosphate	-	-	0.00
Sodium chloride (NaCl)	-	-	0.00
Premix	-	-	0.00

DM, dry matter.

[1] Concentrate comprised of 49.1% of corn grain, 9.7% of wheat bran, 14.6% of cottonseed meal, 19.9% of soybean meal, 2.4% of stone powder, 0.7% of calcium hydrogen phosphate, 1.2% of sodium chloride (NaCl), and 2.4% of premix (on a dry-matter basis).

Source: *Feeding Standard for Beef Cattle* [12].

Supplementary Table S2. Calculation of dietary allowance for the cattle used in Exp 1

Item	HC	LC
Body weight[1] (kg)	227.5	265.5
Target daily body-weight gain (kg/d)	1.0	1.0
Energy requirement[2]		
Net energy for maintenance (NEm, MJ/d)	18.86	21.17
Net energy for gain (NEg, MJ/d)	11.15	12.51
Net energy for maintenance and fattening (NEmf, MJ/d)	31.03	35.27
Correction factor (F)	1.034	1.047
Dry-matter intake[2] (kg/d)	6.00	6.58
Composition of experimental diet (% DM)		
Alfalfa hay	30.0	43.3
Wheat straw	30.0	43.3
Concentrate[3]	40.0	13.3
Net-energy concentration of experimental diet[4] (MJ/kg)	8.59	6.08
Crude-protein concentration of experimental diet[4] (%)	13.07	9.69
Total allowance (estimated, kg-DM)	3.61	5.79
Allowances of feed ingredients (kg-DM)		
Alfalfa	1.08	2.51
Wheat straw	1.08	2.51
Concentrate[3]	1.45	0.77

DM, dry matter; HC, cattle fed high-concentrate diet; LC, cattle fed low-concentrate diet.

[1] The body weight at the time of calculating the daily allowance was used. Therefore, according to the body-weight at the start of Exp 2, the allowances were adjusted.

[2] Calculated on the basis of estimation equation presented in *Feeding Standard for Beef Cattle* [12].

[3] Concentrate comprised of 49.1% of corn grain, 9.7% of wheat bran, 14.6% of cottonseed meal, 19.9% of soybean meal, 2.4% of stone powder, 0.7% of calcium hydrogen phosphate, 1.2% of sodium chloride (NaCl), and 2.4% of premix (on a dry-matter basis).

[4] Calculated on the basis of energy and crude-protein concentrations of feed ingredients in Supplementary Table S1.

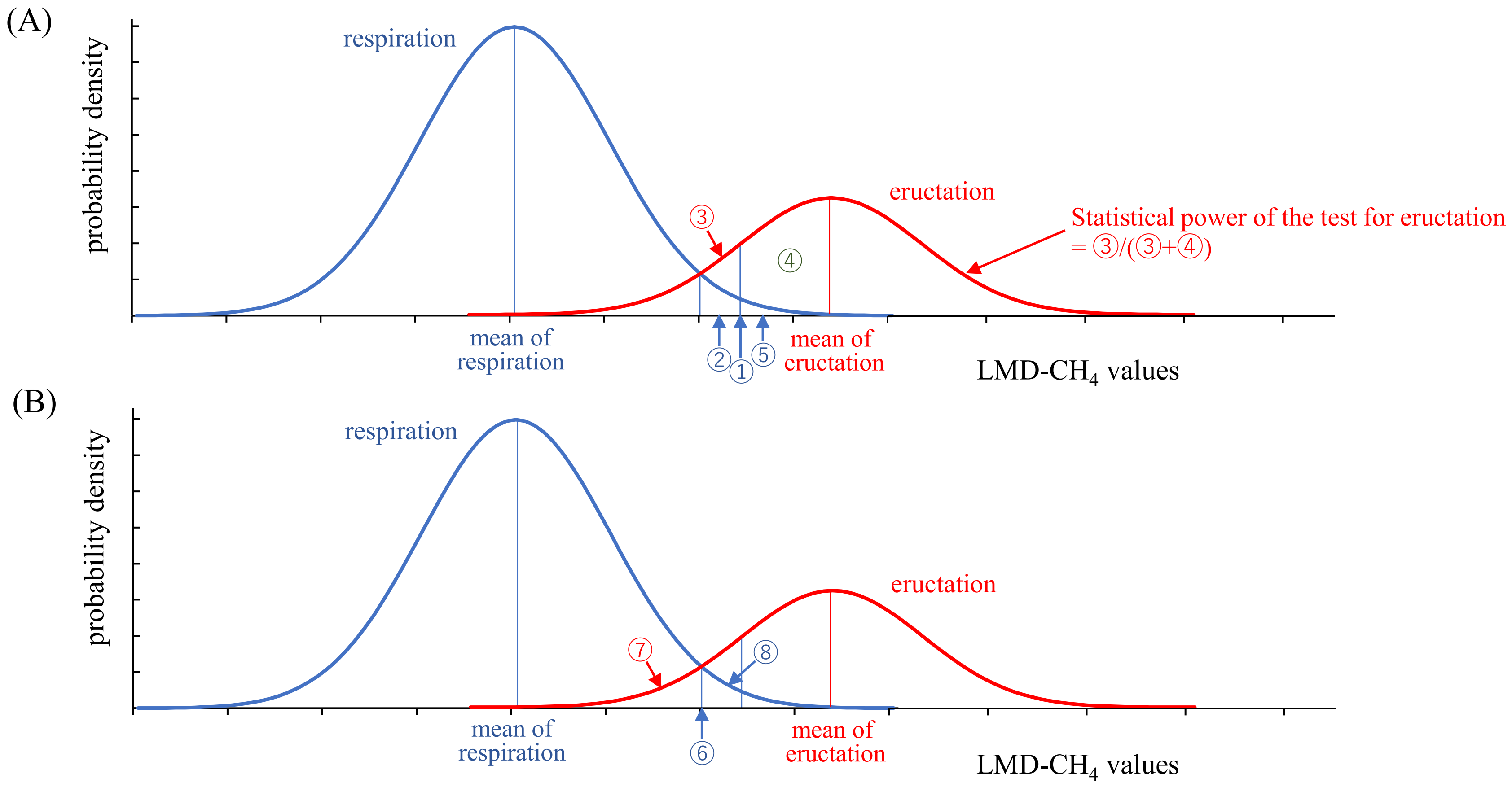

Supplementary Figure S1. Categorization of each LMD-CH_4 value into the value in the normal distribution for respiration or the value in the normal distribution for eructation. A previous report [9] mentioned the threshold at the LMD-CH_4 value with 99% cumulative probability for respiration (①) for separating each LMD-CH_4 value into the value for respiration or for eructation. That is, the area ⑤ shares 1% of total probabilities for respiration. However, in this threshold, it was expected that more values (datapoints) were categorized into respiration part (because the area ③ > the area ⑤) (Figure A). For example, in this method, the value ② are categorized as respiration, though the probability (likelihood) is higher for eructation than for respiration. Therefore, in the current study, we set the threshold so that all the LMD-CH_4 values were categorized into the part for which the values had a higher probability. By setting the threshold at the point ⑥, the statistical errors for both normal distributions would be equal (⑦ = ⑧), and the sum of these two statistical errors (⑦ + ⑧) could be minimized (Figure B).
